# The Brightness of Colour

**DOI:** 10.1371/journal.pone.0005091

**Published:** 2009-03-31

**Authors:** David Corney, John-Dylan Haynes, Geraint Rees, R. Beau Lotto

**Affiliations:** 1 UCL Institute of Ophthalmology, London, United Kingdom; 2 Bernstein Centre for Computational Neuroscience Berlin, Berlin, Germany; 3 UCL Institute of Cognitive Neuroscience, London, United Kingdom; 4 Wellcome Trust Centre for Neuroimaging, University College London, London, United Kingdom; Indiana University, United States of America

## Abstract

**Background:**

The perception of brightness depends on spatial context: the same stimulus can appear light or dark depending on what *surrounds* it. A less well-known but equally important contextual phenomenon is that the *colour* of a stimulus can also alter its brightness. Specifically, stimuli that are more saturated (i.e. purer in colour) appear brighter than stimuli that are less saturated at the same luminance. Similarly, stimuli that are red or blue appear brighter than equiluminant yellow and green stimuli. This non-linear relationship between stimulus intensity and brightness, called the Helmholtz-Kohlrausch (HK) effect, was first described in the nineteenth century but has never been explained. Here, we take advantage of the relative simplicity of this ‘illusion’ to explain it and contextual effects more generally, by using a simple Bayesian ideal observer model of the human visual ecology. We also use fMRI brain scans to identify the neural correlates of brightness without changing the spatial context of the stimulus, which has complicated the interpretation of related fMRI studies.

**Results:**

Rather than modelling human vision directly, we use a Bayesian ideal observer to model human visual ecology. We show that the HK effect is a result of encoding the non-linear statistical relationship between retinal images and natural scenes that would have been experienced by the human visual system in the past. We further show that the complexity of this relationship is due to the response functions of the cone photoreceptors, which themselves are thought to represent an efficient solution to encoding the statistics of images. Finally, we show that the locus of the response to the relationship between images and scenes lies in the primary visual cortex (V1), if not earlier in the visual system, since the brightness of colours (as opposed to their luminance) accords with activity in V1 as measured with fMRI.

**Conclusions:**

The data suggest that perceptions of brightness represent a robust visual response to the likely sources of stimuli, as determined, in this instance, by the known statistical relationship between scenes and their retinal responses. While the responses of the early visual system (receptors in this case) may represent specifically the statistics of images, post receptor responses are more likely represent the statistical relationship between images and scenes. A corollary of this suggestion is that the visual cortex is adapted to relate the retinal image to behaviour given the statistics of its past interactions with the sources of retinal images: the visual cortex is adapted to the signals it receives from the eyes, and not directly to the world beyond.

## Introduction

Brightness has been defined as the perceived intensity of a visual stimulus, irrespective of its source. Lightness, on the other hand, is defined as the apparent brightness of an object relative to the object's reflectance. Thus increasing the intensity of light falling on an object will increase its apparent *brightness* but not necessarily its apparent *lightness*, other things being equal [1]. Saturation is a measure of the spectral “purity” of a colour, and thus how different it is from a neutral, achromatic stimulus. Hue is the perception of how similar a stimulus is to red, green, blue etc. Luminous efficiency, or luminosity, measures the effect that light of different wavelengths has on the human visual system. It is a function of wavelength, usually written as *V*(*λ*) [2], and is typically measured by rapidly alternating a pair of stimuli falling on the same area of the retina; the subject alters the physical radiance of one stimulus until the apparent flickering is minimised. Thus luminance is a measure of the intensity of a stimulus given the sensitivity of the human visual system, and so is integrated over wavelength [3]. Luminance is thought to be used by the brain to process motion, form and texture [4].

Clearly, brightness is monotonically related to luminance in the simplest case: the more luminant the stimulus is, the brighter it appears to be. However, the Helmholtz-Kohlrausch (HK) effect shows that the brightness of a stimulus is not a simple representation of luminance, since the brightness of equally luminant stimuli changes with their relative saturation (i.e. strongly coloured stimuli appear brighter than grey stimuli), and with shifts in the spectral distribution of the stimulus (e.g. ‘blues’ and ‘reds’ appear brighter than ‘greens’ and ‘yellows’ at equiluminance) [1]; [5]–[6].

**Figure 1:**
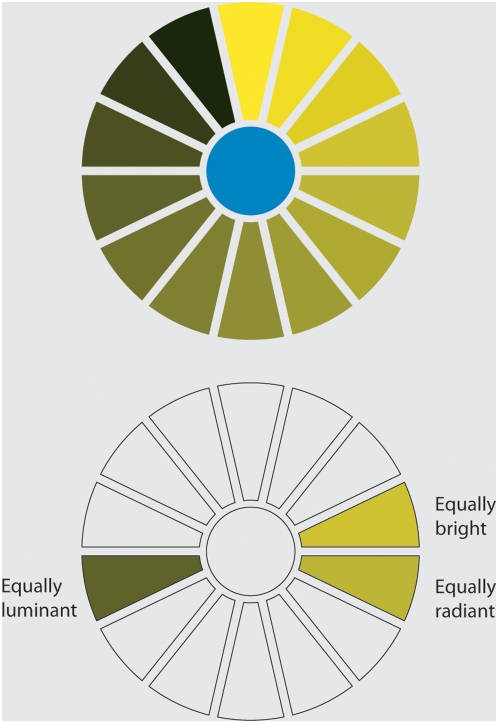
Demonstration stimulus for the Helmholtz-Kohlrausch effect. *Top*: Example of a stimulus used to measure the relationship between reflectance, luminance and brightness. *Bottom*: The wedge most frequently selected by subjects as the best match to the brightness of the blue dot. The wedges that are equally radiant and equally luminant with the blue dot are also indicated.

The HK effect has been measured in a variety of psychophysical studies [7]–[8] and is often expressed in terms of the (variable) ratio between brightness and luminance. A simple example of this phenomenon is shown in Figure 1. The upper panel shows a blue dot encircled by yellow wedges of various intensities. The lower panel in the same figure shows the yellow wedge that observers typically chose as being “equally bright” to the blue dot, and the wedges that are equally luminant and equally reflective (and therefore equally radiant under a constant light) as the dot. (Note that the specific measures of luminance and radiance will depend in part on the nature of the display; the observers in this case were shown a paper copy under natural daylight illumination.) An important aspect of the HK effect – that is usually overlooked – is the asymmetry in the wavelength-dependency of the effect. Specifically, short wavelength light (blue) appears brighter than equiluminant long wavelength light (red) [9].

Together, these observations demonstrate that the shape of a stimulus' spectral distribution (in addition to its spatial information) can be described as providing a ‘context’ for its perceived intensity. While such spectral ‘contextual effects’ on the brightness of a stimulus have been accurately modelled from colorimetric information (e.g. by using the Ware-Cowan equations [1, p. 142]), there remains no clear explanation for this phenomenon [9].

Previously, it has been argued that *spatial context* alters the brightness of a stimulus because the visual system has adapted to the complex statistical relationship between images and scenes experienced previously [10]–[12]. While proving to be a useful framework for rationalising illusions of brightness and the like, a problem with this framework is that the visual experience of individuals (with regard to surface reflectance and illumination) and that of their evolutionary ancestors is unknown if not unknowable. (For a counter-example see ref. [13] with quantitative explanations of perceived orientation.) To overcome this limitation, one recent strategy has been to replace the human observer with a simulation, such as an artificial neural network [14]–[15] or a Bayesian ideal observer [16]. This can then be embedded in an ecologically-relevant synthetic environment, where experience can be perfectly controlled and behaviours unambiguously measured. Using this strategy, we have previously shown that a variety of brightness illusions represent a robust response to the statistical relationship between images and scenes, independent of specific features of the visual system itself [15] (see also ref. [17] for a complementary explanation for illusions of space).

More generally, ideal observer analysis provides a principled approach for understanding natural tasks including vision. An ideal observer is a model that performs a given task in an optimal way, limited only by the information available and explicitly specified constraints [16]. They have been used to provide insight into a number of vision science problems, such as comparing measurements of primate photoreceptors with receptors that are “ideal” for detecting fruit amongst foliage [18]; analysing human performance at an image classification task [19]; and modelling the colour appearance of small spots [20].

Here, we build on this computational approach and combine the ideal observer model with the known human cone sensitivities, thus incorporating what would have been the statistical mapping from scenes to post-receptoral neural signals in the past. The aim in doing so is not to model human perception explicitly, but to determine whether human perception – in this case the HK effect – represents a robust solution to the relationship between scenes and images (retinal responses) that must have been experienced by the primary visual cortex during evolution.

## Results

A scene is the physical structure of the world as described by the spectral properties of light emitted or reflected from objects in the world. We define an image as the retinal photoreceptor response to light that reaches the eye, and perception as the conscious or behavioural response to the image. In the experiments described below, we first build a simple model of natural scenes, retinal responses (‘images’) and predicted reflectances (‘perception’), and show that it corresponds to human perception of the brightness of colours. We then alter first the statistics of scenes; second the retinal response functions (and hence the statistics of “natural” images); and third the relationship between scenes and images; and in each case measure the effect on predictions of reflectance by the ideal observer.

### Modelling scenes and retinal responses

Here we describe our model of scenes and images (independent of perception) that will be used as the basis for modelling the source of the HK effect. We begin with the proportion of light that a surface reflects at different wavelengths, which is described by that surface's spectral reflectance function (SRF). For natural surfaces, SRFs tend to vary smoothly [21]–[22] probably due to the absorption patterns of individual molecules within the surface that are superimposed on each other by molecular interactions [21]. Previous models combining as few as five suitable basis functions have been shown to accurately model natural surfaces [21], [23]. Therefore, to produce synthetic SRFs that are naturalistic while also being relatively straightforward to control and interpret, we use a mixture of Gaussian basis functions. The value of a single Gaussian function *G*, at any given wavelength *λ*, is characterised by its mean (*μ*), variance (*σ* ^2^) and amplitude (*α*):
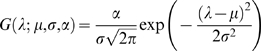



By mixing a number of Gaussians together, more complex and realistic SRFs can be defined:
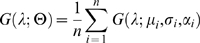
where *Θ* is the set of model parameters defining *n* Gaussian components. To test the natural relevance of this model, we fitted such a mixture of Gaussians to each of a sample of pixels from hyperspectral images of natural scenes. These are rural scenes, principally vegetative, filmed under clear skies in direct sunlight [24]. We use the published estimated reflectance spectra of random portions of the scenes. Using three Gaussian components fitted using a standard least-squares optimisation procedure, this analysis resulted in *r*-squared scores in the range 0.91–0.96, demonstrating that natural surface reflectances are indeed accurately modelled in this way. (See Methods, “Fitting Gaussians to hyperspectral data” for details.)

To complete our model of natural scenes, we model the illumination of the synthetic surfaces just described with natural daylight, as defined by the CIE “standard illuminant” D65. To calculate this, at each wavelength *λ*, we multiply the value of the SRF by the proportion of D65 at that wavelength. Thus, each of our scenes is a synthetic SRF under D65 illumination, producing an emitted spectral power distribution (SPD).

To model the resulting *retinal response* arising from each scene (i.e. a synthetic surface under D65 illumination) we calculate the response vector ***c*** for long-, medium- and short-wavelength human cones: 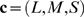 [25]. (See Methods, “Retinal responses” for further details.) Luminous efficiency, or luminosity is defined as *V* = 0.64315*L+0.39595*M [2]. This is the sum of long- and medium-wavelength cone activity, weighted in proportion (approximately) to their existence in the retina [3].

Finally, in this model we assume that brightness is the visual system's estimate of the amount of light reflected from a surface. With a constant source of illumination, this represents the estimate of reflectance, and is therefore proportional to 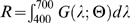. This is the area under the mixture of Gaussians that define the SRF, and is the total proportion of incident light that is reflected by the surface. For the rest of this paper, we define a surface's brightness as the area under its SRF, and ignore the scaling constant.

A set of such scenes and retinal responses defines a ‘training set’ that is used to optimise the Bayesian ideal observer model described below.

### Modelling the ‘psychophysical’ test set

While the spectra used for the ‘training set’ are consistent with natural spectra, the spectra used for measuring the ideal observer's ‘predicted reflectance’ of surfaces (the ‘test set’) are the much simpler spectra used in psychophysical experiments for measuring the HK effect in humans. Here, each stimulus is defined by a narrow-band spectral power distribution (SPD), consisting of a single rectangle whose dominant wavelength, width and height correspond to the mean, variance and amplitude of the Gaussian spectra described above. This can be thought of as the emission of an idealised xenon arc lamp, filtered to produce a square cut-off, as is commonly used in traditional colorimetric studies. A set of 10,000 such spectra was used covering the whole range of monochromatic wavelengths, with different intensities and saturations.

In order to determine whether the HK effect is a robust response to the non-linear relationship between images and scenes, it is necessary to relate the qualities of surfaces and images to their perceptual qualities (i.e. their hue, saturation and brightness). This must be done in a way that is independent of the observer to avoid hidden biases in the results. By limiting our test set to simple Gaussian spectra, the physical correlate of hue is readily defined as the wavelength corresponding to the peak of the training/test spectrum, and the physical correlate of saturation as the *standard deviation* of the intensity of the spectral power distribution of the training/test spectrum. The latter gives a score of zero for uniform stimuli – corresponding to human percepts of grey, black and white – and larger scores for SPDs with “narrower” peaks. Note that this measures the deviation of the *intensity* of the SPD around the mean intensity, and not the deviation of the *wavelength* around its mean. The variance of the wavelength is a parameter of the Gaussian functions described above. (See Methods, “Physical correlates of colour properties” for further details.)

### Bayesian ideal observer analysis

An ideal observer is a theoretical model that performs a task as well as is theoretically possible, relative to the available information and any specified constraints. Any Bayesian ideal observer works in several stages [16]. First, it computes the likelihood function, i.e. the distribution of the target variable given the observed variables. Second, it convolves this likelihood function with the prior probability distribution of the target variable(s) to get a function proportional to the posterior distribution. Third, this posterior is convolved with the utility function. And finally, the maximum of this function is identified as the optimal prediction of the target variable(s). In the model here, the task of the ideal observer is to predict the reflectance of a previously unseen surface (‘scene’) given only the corresponding cone response functions (‘image’) and a finite set of previous observations (‘experience’). We assume that all types of prediction errors are equally costly, so the utility function can be ignored. Thus, our ideal observer must calculate the value of *R* that maximises the product of the probability of observing the cone activations given the reflectance *R*, and the prior probability of *R*: 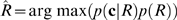
(1)where 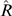 is the optimal estimation of *R*. By the definition of conditional probability, this is identical to
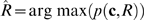
(2)where we use the joint distribution of ***c*** and *R* for convenience. Thus 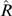, the estimate of the physical correlate of brightness, is the value of *R* that maximises *p*(**c**,*R*) for the observed cone activations **c**. Any errors, i.e. any discrepancy between *R* and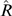, must be due to insufficient information contained in the cone responses ***c***, or insufficient data in the sample of previous observations used to estimate the distributions. One such real-world example is metamerism, when two distinct surfaces produce identical cone responses under some particular illuminant and are therefore indistinguishable, even if they are easily distinguishable under some other illuminant [24].

A dataset of 25,000 distinct scenes (surfaces under D65 illumination) and their corresponding images was generated. This dataset defined the full history of ‘previous observations’ of the ideal observer, and therefore characterises the visual environment to which the observer is adapted. Surface SRFs were generated by an equal mixture of three Gaussian functions with uniform distributions of μ, σ and α (ranges: 100≤μ≤1000; 1≤σ≤100; 1≤α≤100). In this way ‘scene space’ was uniformly sampled, which means the prior probability for all surfaces is equal. Furthermore, the cone response functions ***c*** and the surface reflectance *R* (scenes) together define the joint probability distribution in equation (2).

To predict the reflectance corresponding to a novel SPD given the corresponding three cone responses, the ideal observer simply searches through the 25,000 ‘previous observations’ to find the reflectance that generated the most similar pattern of cone responses. Here, ‘most similar’ is defined as the shortest Euclidean distance between the training and novel images, in terms of the cone response vectors, ***c***. Given the available information, and without making further assumptions, this is guaranteed to return the most likely 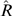 estimate in equation (2). While a more sophisticated model could interpolate between several close examples, such as with a *k*-nearest neighbour classifier, any such interpolation would require making further assumptions about the statistics of scenes, stimuli and perception. Conversely, this model cannot reliably extrapolate in response to novel stimuli beyond its past experience. However, as long as the test images used are within the broad distribution of training experiences, this will not affect the results. Note also that the model does not incorporate adaptation in response to each stimulus' brightness or colour. This is equivalent to giving only brief stimulus presentations to a human observer.

To determine the accuracy of the ideal observer, we set the ideal observer a simulated ‘psychophysical’ test composed of 10,000 novel scenes (created as described above) and their corresponding images. The root-mean-squared (RMS) error between the predicted and true reflectance was then calculated, after both had been standardised to the range 0–1. The resulting RMS error was 0.099. This only slightly improved with much larger training sets, presumably due to metamerism as discussed above. Thus, the ideal observer was able to accurately predict the reflectance (which we take to be the physical correlate of brightness) of a novel spectral stimulus given only a finite set of known ‘experiences’.

### Measuring the HK effect in the Bayesian Ideal Observer

**Figure 2:**
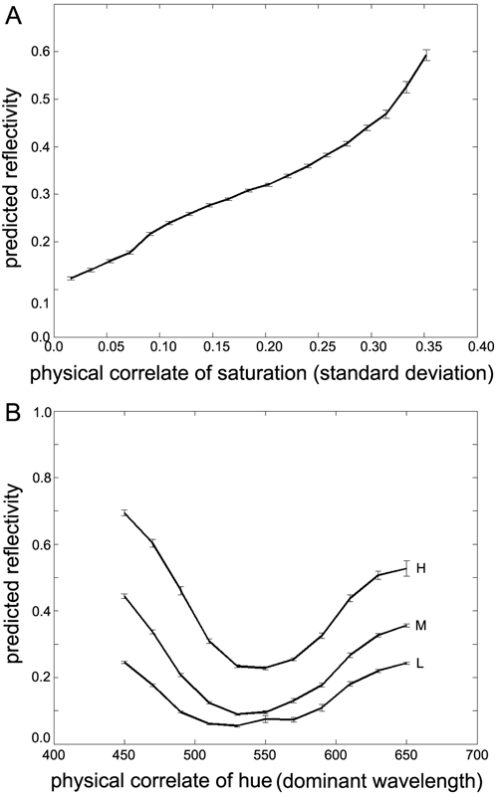
The Bayesian ideal observer predictions of reflectance. A) Predictions of reflectance as *σ* (the physical correlate of saturation) varies, for a constant luminance. Low saturation surfaces lead to low predictions of reflectance, and high saturation to high predictions of reflectance. Error bars indicate 1 standard error. B) Predictions of reflectance as *μ* (the physical correlate of hue) varies, for a constant luminance. The ideal observer predictions of reflectance for different dominant wavelengths, grouped by low (L, 0.013), medium (M, 0.051) and high (H, 0.14) luminance values. This shows that for any given luminance, yellows and greens are seen as less reflective than blues and reds. Error bars indicate 1 standard error.

We next investigate the first feature of the HK effect. Specifically, we ask whether the ideal observer shows a positive correlation between brightness and saturation for equiluminant stimuli – like humans. The test set of 10,000 novels scenes and images was again presented to the ideal observer. Figure 2A plots *σ* (the physical correlate of saturation) against 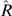 (the physical correlate of brightness) for a constant luminance. Clearly, increasing the physical correlate of saturation of a stimulus increases the ideal observer's predicted reflectance of that same stimulus (correlation coefficient *r* = 0.992, *p*<10^−12^), consistent with human perception.

We next considered the second major aspect of the HK effect. Specifically, we ask whether the ideal observer also shows a relationship between hue and brightness of equiluminant stimuli that is qualitatively similar to humans. In other words, does the ideal observer predict the reflectance of ‘red’ and ‘blue’ stimuli to be greater than equiluminant ‘yellow’ or ‘green’ stimuli? Using the same 10,000 novel scenes and corresponding stimuli as in the experiment just described, we plot in Figure 2B the predicted reflectance, 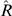, for groups of samples of constant luminance arranged according to dominant wavelength. Each line corresponds to a constant luminance from 0.011 (‘low’; bottom line) to 0.152 (‘high’; top line), chosen to represent the quartile intervals of the range of luminance scores in the test set. As expected, the three levels of luminance are clearly separated as three levels of predicted brightness, with high-luminance stimuli corresponding to bright surfaces. Note, however, that each line also shows a clear dip around 550 nm, corresponding to a lower predicted reflectance for middling wavelengths (greens and yellows) compared to wavelengths at the ends of the spectrum (reds and blues), which again is consistent with human perception. Furthermore, the predicted reflectance of the ‘blue’ surfaces is higher than that for red surfaces, which is a more subtle and less reported aspect of the HK effect that is also present in human measurements [9].

Taken together, these results show a strong qualitative similarity between our Bayesian ideal observer and human perception vis-à-vis the HK effect. This suggests that the HK effect exists in humans in part because human perception is adapted to the complex statistical relationship between scenes and images, and not, for example, due to particular brain physiology. We next sought to determine the underlying cause of this statistical relationship between images and scene to which the visual system has adapted.

#### The effect of scene statistics

We now consider whether the HK effect represents the frequency of occurrence of different surface colours (i.e. is a direct consequence of specifically the *statistics of scenes*). For example, if ‘blue’ and ‘red’ surfaces were experienced more often, then it could be argued that the visual system should adapt directly to these statistical regularities and perceive red and blue surfaces to be brighter than yellow and green surfaces at equiluminance. To investigate this, we generated new training sets by varying the distribution of *μ* (the physical correlate of hue) used to generate the mixture of Gaussians defining the SRF. We then tested the resulting ideal observer models on the same “psychophysical” test set used above.

**Figure 3:**
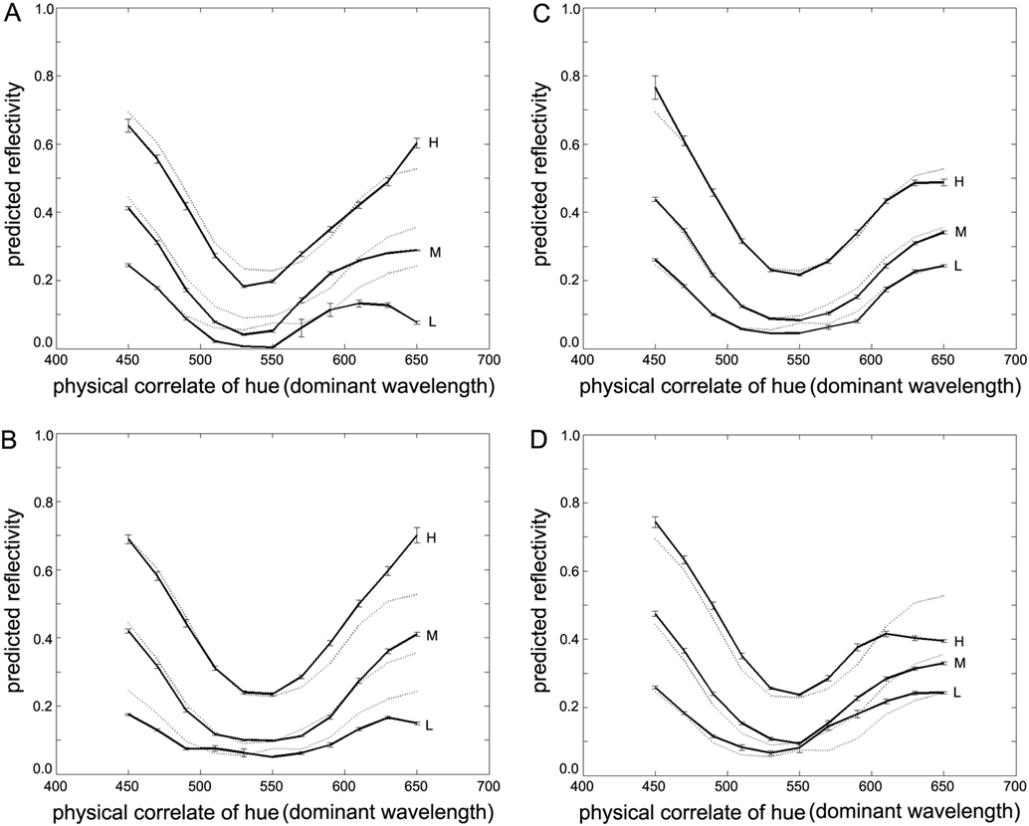
Ideal observer predictions when training scenes with altered scene or image statistics. A) Ideal observer predictions when training scenes are biased towards “green”. The training set of SRFs is a mixture of 3 Gaussians as before, but here one component is forced to be centred at 550 nm, which typically appears green. For comparison, the previous “default” results from Figure 2B are shown using dotted lines. B) Ideal observer predictions when training scenes are biased towards “red” and “blue”. The training set SRFs are still a mixture of 3 Gaussians as before, but here one component is forced to be centred at either 450 nm (“blue”) or 650 nm (“red”) for each surface. For comparison, the previous “default” results are shown using dotted lines. C) Ideal observer predictions with increased M *λ*-max. The M-cone peak sensitivity is now 560 nm on both training and test sets, instead of the usual 545 nm. For comparison, the previous “default” results are shown using dotted lines. D) Ideal observer predictions with decreased M *λ*-max. The M-cone peak sensitivity is now 505 nm on both training and test sets, instead of the usual 545 nm. This is mid-way between L-cone and S-cone *λ*-max values. For comparison, the previous “default” results are shown using dotted lines.

We first introduced a bias towards mid-wavelength surface reflectance functions by forcing one of the three component Gaussians to have a centre (*μ*) of 550 nm. The remaining two components had random means as with the experiments described above. This simple change in the distribution of training surfaces produces a new ideal observer that has still experienced a wide range of SRFs, but has experienced some hues much more often than others. Figure 3A shows the resulting predictions of reflectance as the test hue varies, for constant luminance scores. Although there is some change, the overall pattern is much the same, namely that there is a large drop in predicted reflectance around the centre of the visual spectrum.

In a second training set, the distribution was altered making reds and blues *more* common. In half the records, one Gaussian component was forced to have a centre at 450 nm, and in the other half, it was forced to be at 650 nm. In all cases, the remaining two Gaussian components had random centres, and all widths and amplitudes were random, as before. Despite this change in the distribution of *μ*, the predicted reflectances of SRFs with dominant wavelengths in the middle of the visible range remained lower at equiluminance (Figure 3B), and reflectance remained correlated with saturation (*r* = 0.98; *p*<10^−12^).

We conclude that the HK effect of the ideal observer is largely independent of the distribution of the dominant wavelength in the historical training sample, i.e. it is independent of this particular scene statistic. In both of the experiments just described the correlation between saturation and predicted reflectance was still significantly positive (*r*≈0.98; *p*<10^−13^).

### The effect of retinal response statistics

For a given scene, the image that it generates in the eye depends on the nature of the photoreceptors. By adjusting this aspect of our model, we can ask to what extent the specific features of the (human) cone response functions – as incorporated into our ideal observer model – are responsible for the different aspects of the HK effect (i.e., the statistics of images). Unlike psychophysical studies on humans, we are of course free to alter the number of cone types and their peak sensitivities (*λ*-max) in the synthetic world. One parallel in the natural world is the presence of oil droplets in the cone cells of many species of birds, which act as long pass filters [26] and effectively alter the cell's peak sensitivities. One constraint is that luminosity is defined in terms of observed long- and medium-wavelength sensitive cone responses (L- and M-cones); we therefore limit ourselves to three-cone models in these experiments.

To alter the formation of images in our model, we adjusted the medium-wavelength cone sensitivity function to have a different peak sensitivity, without changing the overall shape of the function. The default *λ*-max values for the L-, M- and S-cones are 570, 545 and 445 nm respectively, based on humans [25]. We first increase the M-cone *λ*-max from 545 nm to 560 nm, and then in a second experiment, decrease it to 505 nm (which is halfway between the L- and S-cone peaks). The L- and S-cone sensitivities remain unchanged throughout. We use these new cone sensitivities to define the ideal observer's images for both the training set and the test set. Figure 3C shows the results for an M-cone *λ*-max of 560 nm and Figure 3D shows the corresponding results for an M-cone *λ*-max of 505 nm. Both cases show that even a substantial shift in the M-cone response function has little effect on the predicted reflectance of the stimulus: the HK effect remains. In both of these cases, the correlation between saturation and predicted reflectance is again significantly positive (*r*≈0.97; *p*<10^−10^).

Together, then, the experiments demonstrate that changing either the *scene statistics* or the *image statistics* does *not* fundamentally change the ideal observer's Helmholtz-Kohlrausch effect. This suggests that the HK effect in humans is unlikely to be caused by such aspects of the visual ecology or visual system. We now investigate whether the HK effect arises from the *relationship between* scenes and images.

### The effect of the relationship between scene and retinal response statistics

We train an ideal observer on a data set with one relationship between scenes and images, and then test it on a new data set with a different relationship. More specifically, we train the model using filters to vary the intensity of incident light at specific wavelengths. This experiment can be thought of as modelling the effect of wearing “purple” (green-absorbing) contact lenses for an extended period of time (allowing for adaptation at all stages of the visual system), and then removing them and testing the resulting patterns of perception before any “re-adaptation” can take place.

**Figure 4:**
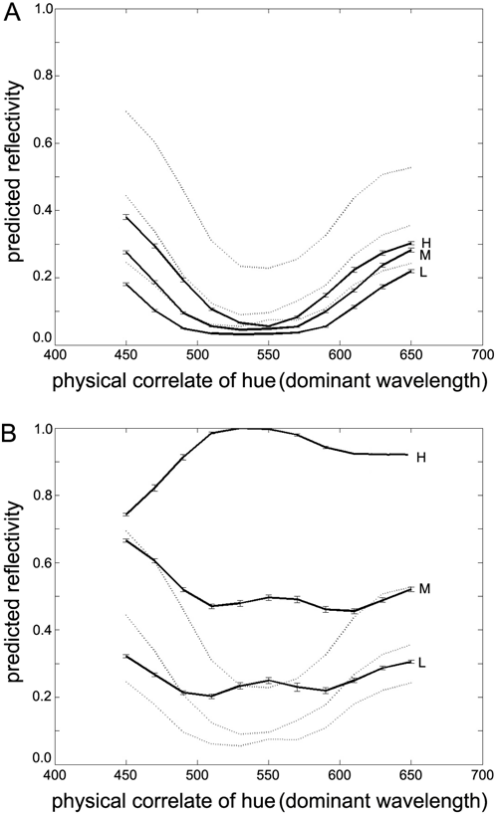
Ideal observer predictions with a green-absorbing filter. A) Ideal observer predictions when trained with a green-absorbing filter and tested with the same filter on the standard test set. For comparison, the previous “default” results are shown using dotted lines. The predictions are now smaller, but follow the same overall pattern. B) Ideal observer predictions when trained with a green-absorbing filter and tested on the “unfiltered” standard test set. For comparison, the previous “default” results are shown using dotted lines. The predictions now follow a very different pattern.

First, we introduce a coloured filter to the ideal observer. This reduces light intensity in the middle of the visible spectrum to a degree specified by a Gaussian function centred at 550 nm. Averaged across many scenes, this filter transmits around 80% of light at 450 nm and 650 nm and just 5% at 550 nm. If the same filter is used during training *and testing*, then there is little effect as Figure 4A shows, except for an overall reduction in the predicted reflectivity. The characteristic dip in the middle of the spectrum remains. However, if this “purple” filter is removed and the same ideal observer is re-tested, then its behaviour changes considerably, as shown in Figure 4B. The most obvious effect is that the predictions of reflectivity are now typically higher: removing the filter means that more light is reaching the photoreceptors overall, so each scene appears lighter than before. More importantly, however, Figure 4B also shows a considerable “flattening” of the range of responses, suggesting a substantial reduction in the strength of this aspect of the HK effect.

To quantify the degree of this flattening, we can calculate the sample standard deviation of the predicted reflectance scores for each experiment. The standard deviations for the initial experiments, as shown in the three curves of Figure 2B, are 0.0750, 0.1209 and 0.1580 for the low, medium and high luminance cases respectively. The average equivalent scores across the previous experiments (i.e. the results shown in Figures 2B and 3) are 0.0689 (±0.023), 0.1159 (±0.023) and 0.1542 (±0.044). For this final case (Figure 4B) the scores are 0.0387, 0.0650, and 0.0784 respectively, showing that the predicted reflectances vary much less with wavelength (about half as much) in this final experimental case. As before, the correlation between saturation and predicted reflectance was still significantly positive (*r*≈0.95; *p*<10^−13^).

Thus changing the statistical relationship between the scenes and images (the retinal responses), by introducing a filter “in front of” the receptors during training and then removing it, leads to a fundamental change in brightness. We therefore conclude that the HK effect in humans is a direct consequence of adapting – presumably in post-receptor processing – to the statistical relationship between images and their source scenes.

### Colour brightness in the brain

Where in the brain might this ‘adaptation’ to the statistical relationship between luminance and surface reflectance (our physical correlate of brightness) take place? Previous experiments have suggested that activity in V1 is correlated with brightness [27]–[31]. This implication, however, is inconclusive because the test stimuli used in the relevant studies usually differed in spatial contrast, which is more highly correlated with human V1 activity than brightness is [29]. On the other hand, the HK effect affords the opportunity to vary brightness *without* varying either luminance or spatial contrast.

**Figure 5:**
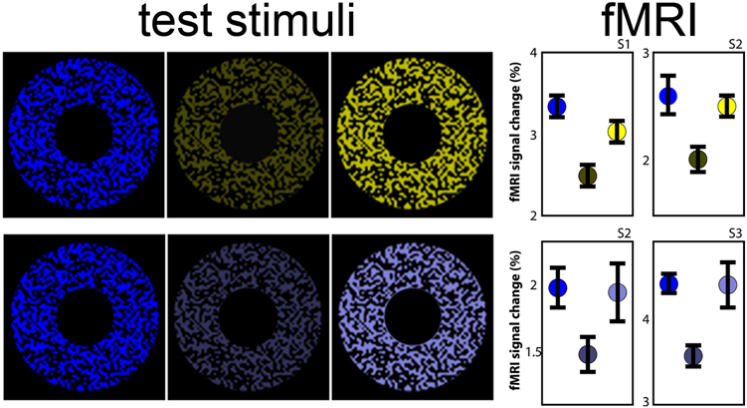
fMRI experiments: stimuli and results. *Top row*: The blue standard (left), equiluminant yellow (middle), and equally bright (right) annuli used in the first fMRI experiment; the relative responses (percent signal change) of primary visual cortex (V1) to the these stimuli (in the same left-to-right order as shown in the left panels) are shown on the right for two subjects (S1, S2). Error bars indicate one standard error. *Bottom row*: Blue standard (left), equiluminant low-saturated blue (middle), and equally bright low-saturated blue (right) annuli used in the second fMRI experiment; again the relative response of primary visual cortex (V1) of two subjects to these stimuli are indicated on the right (S2, S3). Display conventions are as for the upper row. Evidently V1 activity is consistent with the probable image-source relationship rather than the characteristics of the stimuli as such.

Subjects were presented with the three coloured annuli shown in Figure 5: (1) a blue ‘standard’ annulus having the maximum saturation and luminance generated by a typical LCD projector; (2) an equiluminant saturated yellow annulus; and (3) an equally saturated yellow annulus whose intensity could be adjusted by each participant to match the brightness of the blue standard. All stimuli were viewed against the same black surround, making chromaticity the only contextual parameter to luminance (luminance being determined by spectrophotometric measurement). In a further experiment, the yellow annuli were replaced with less-saturated blue annuli that were either equiluminant with, or seen as being equally as bright as, the saturated blue annulus.

The retinotopic responses in V1 to each different annulus in Figure 5 were measured using high-field functional MRI (see Methods, “fMRI acquisition”). The retinotopic V1 response evoked by the saturated blue annulus was significantly greater than that evoked by the equiluminant yellow (Subject 1: T9 = 14.77; *p*<0.0001; Subject 2: T9 = 3.71, *p* = 0.0024) or less-saturated blue annuli (Subject 2: T4 = 2.94, *p* = 0.0212; Subject 3: T3 = 4.5814, *p* = 0.0098), as shown on the right of Figure 5. In contrast, the cortical responses to annuli matched for brightness were nearly identical, despite the physical difference in their luminance (an identical pattern of responses was also found in area V4; data not shown).

The responses of human V1 therefore more closely reflected the brightness than the physical luminance. There has been little previous study of how V1 responses to chromatic stimuli vary as a function of luminance, although V1 shows robust responses to chromatic stimuli *per se* [32]. We cannot therefore entirely rule out the possibility that such a close correspondence between brightness of chromatic stimuli and V1 activity represents a coincidentally identical response to two different hue-luminance response functions. Nevertheless, taken together with the empirical work presented here, the most parsimonious explanation is that responses of V1 correlate with brightness, and thus surface reflectance, suggesting that processing leading up to V1 accommodates the non-linear relationship between images and scenes.

## Discussion

The HK effect, like its close relations – simultaneous brightness contrast, the Hunt effect and the Abney effect – is an example of how the human perception of brightness is not a direct mapping of a light stimulus. Rather, spatial, temporal and spectral context influence the brightness that one perceives. In the case of the Helmholtz-Kohlrausch (HK) effect, more saturated (purer) colours appear brighter than less saturated colours at equiluminance, as do red and blue colours compared to yellow and green colours. This complex relationship between luminance and brightness was first described by Helmholtz but never subsequently explained, despite extensive study. Here we show that it is likely to be an adaptive, robust solution to the problems caused by the complex relationship between images and scenes, which is itself conferred by the spectral sensitivity of the cones. Put simply, red and blue spectral stimuli appear brighter than equiluminant yellow and green stimuli, and more saturated stimuli appear brighter than less saturated stimuli, because the former in both cases would have signified more reflective surfaces in the past. We further suggest that this statistical relationship concerning the different responses to equiluminant stimuli is represented in the functional structure of the human primary visual cortex, where we show activity in V1 is better correlated with brightness than with physical luminance.

### Why should the spectral quality of a stimulus alter its brightness?

There is increasing evidence to suggest that, whatever the mechanisms, natural visual systems evolved to encode the past empirical significance of stimuli [11]–[13]; [33]–[35]. The merits of this view notwithstanding, the principal obstacle for directly testing this hypothesis in humans is the paucity of information about actual visual experiences. Most statistical models of brightness, therefore, focus either on what it is possible to measure (e.g. the statistics of natural images), or make predictions based on assumptions about the physical world, visual experience and/or psychophysical measures of perception itself. However, this limitation can, to a certain degree, be overcome in simpler systems (natural or synthetic) by raising and/or evolving them in highly controlled visual ecologies. This has been successfully achieved with experiments of bumblebees [35] and artificial-life systems [36]. Here we directly explored the empirical basis of human brightness and lightness perception by taking advantage of the fact that the visual system is differentially sensitive to wavelength.

**Figure 6:**
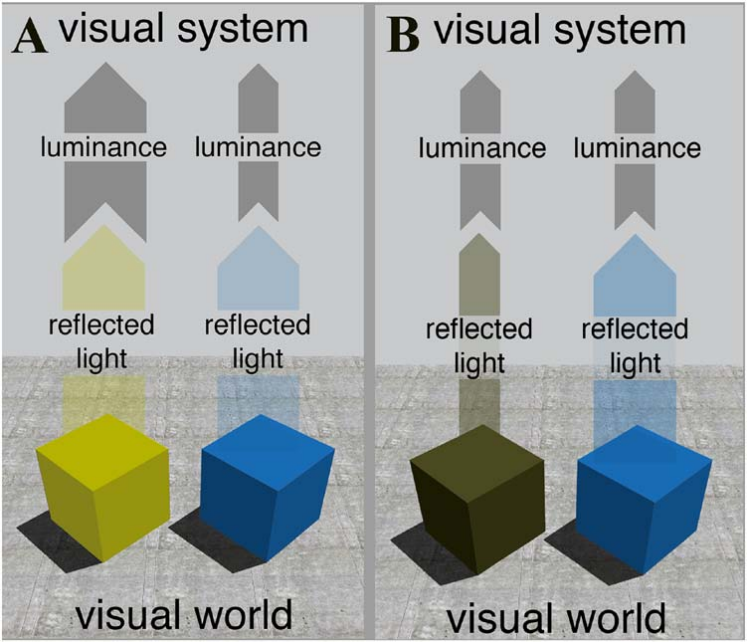
Relationship between luminance, object reflectance and colour percepts. *Left*: The yellow and blue objects reflect the same quantity of light and appear equally bright to observers. However, due to the lower sensitivity of the retina to shorter wavelengths, the stimuli arising from the two objects elicit very different luminance signals. *Right*: A darker yellow object reflects less light but generates the same luminance signal as the blue object. It nonetheless appears less bright than the blue object.

Measures of responsiveness to spectral stimuli (such as cone absorption spectra and tests of threshold detection as a function of spectral composition) all show that the human visual system is less sensitive to long and short wavelength light than to light of middle wavelengths [2], [37]. Previous modelling has clearly demonstrated that the cone response functions [38], [4] and early post-receptor processing [39]–[40] are efficient solutions that maximise the information content of the stimuli that fall onto the eye. The consequence of this efficient coding, however, is that it imposes an inherent bias in early visual processing: surfaces reflecting predominantly long and/or short wavelengths (and thus appear red or blue, respectively), and surfaces that reflect a narrow range of wavelengths (and thus appear more saturated) will on average generate a weaker luminance signal than surfaces that reflect predominantly middle wavelengths (Figure 6). This means that if the visual brain represents scenes according to the similarities among their constituent objects, in addition to efficiently encoding the images each scene generates, then post-receptor processing must explicitly encode the non-uniform sensitivity of its receptors in order to “re-engineer” the probable scene from any image. This could be achieved by weighting the interaction between the luminance channel and the colour opponent channels according to the relationship experienced between the retinal image and reflectance. Indeed, specific weighting functions between these channels have been suggested in previously colorimetric studies, but no ecological rationale for their differential weighting has been offered. We therefore asked whether the HK effect represents an adaptation in post-receptor processing to this known statistical bias, which causes blue and red stimuli to appear brighter than equiluminant yellow or green stimuli, and more saturated stimuli to brighter than less saturated stimuli; and also whether this bias is represented in the activity in the V1, which may itself result from a differential weighting of the luminance and opponent channels.

We tested this hypothesis in two types of experiments. First, we created a simple synthetic model of human visual ecology, and embedded within this ecology an ideal observer. Importantly the HK effect was still present in the ideal observer when the scene statistics were drastically altered (such as changing the frequency of certain colours). Equally, the HK effect of the ideal observer was largely unaltered when statistics of images arising from the synthetic ecology were altered (such as by changing the response functions of the photoreceptors). Second, we showed that changing the statistical relationship between scenes and retinal images *did* change the HK effect for the ideal observer, to the extent that the characteristic relationship between hue and reflectance was greatly flattened (Figure 4B). Because a large fraction of the mid-spectrum light is attenuated by the filter during training, a stronger stimulus is now required at these wavelengths to generate the same luminance. During the subsequent test, without the filter, these same stimuli generate a much greater luminance, reducing or even inverting the characteristic dip in responses seen normally. Thus changing the historical scene/image relationship effectively removes the HK effect, strongly suggesting that it is this relationship that accounts from the HK effect in humans. Note that our model does not attempt to distinguish between evolution and learning. Instead, we model the ‘adaptation’ of the visual system to its environment, irrespective of the timescale involved or the nature of the encoding (such as genetic or neurophysiological).

The other key feature of the HK effect is that more-saturated surfaces appear brighter than equiluminant less-saturated surfaces. To understand this, consider the spectral power distribution (SPD) of the light reflected by a highly saturated “blue” surface. This SPD will have a peak in the short-wavelength region of the spectrum, and will be relatively low elsewhere. Suppose we then decrease the saturation by steadily “whitening” the SPD, while keeping the total energy of the light (as shown by the area under the SPD curve) constant. Recalling that the luminous efficiency function peaks near the centre of the visible spectrum [2], this decreasing saturation can only lead to an *increase* in luminosity. Conversely, if the luminosity is held constant, then a decrease in saturation will correspond to a decrease in the total energy of the SPD; as before, if we assume a constant illuminant, then this can only be caused by a decrease in reflectance.

The exact nature of this correlation between saturation and brightness for equiluminant lights will depend on the specific SPD and on the observer's luminous efficiency function. For SPDs that are most intense in the middle of the visible spectrum (e.g. yellow or green lights), the effect will much reduced, which is consistent with the higher inter-subject variability that has been reported for such stimuli, and even the occasional reversal of the correlation [9]. Nonetheless, when averaged across many equally radiant stimuli with dominant wavelengths across the visible spectrum, the mean correlation between saturation and luminance will be negative as described above. This means that for two equiluminant SPDs, the one with the greater saturation is likely to have been reflected from a surface with greater reflectance than the other. Therefore the human visual system predicts that the more saturated surface is *brighter* than the other surface, as reported in the literature [1], [5], [6], [9] and as shown by our model.

That perceptions of colour are explained by the statistics of past experience with natural scenes has been suggested and tested previously [41]–[42], [12], [15], [21]. Of particular relevance is work that shows that the cumulative density functions of the joint probability distributions between the ‘physical correlates’ of hue, saturation and brightness, predict the HK effect [41]. Consistent with this possibility, the research described here provides an explicit explanation of the HK effect in terms of the inevitable bias in the ‘experience’ of later visual areas in terms of human cone fundamentals, irrespective of how they evolved or what they encode. We further show that this bias - rather than a bias in the distribution of natural spectra or in the cone response functions *per se* - is sufficient to explain the HK effect.

### Where is the statistical relationship between the brightness of images and scenes encoded in the brain?

Next, we investigated the neural correlates of colour brightness. In particular, we sought to determine whether the primary visual cortex represents stimulus luminance or brightness, and, by inference, the probable reflectance of the spectral stimulus. In two high-field functional MRI experiments, three participants with normal vision were presented with three coloured annuli (Figure 5). In both experiments the response to the blue standard was significantly greater than to the equiluminant yellow or unsaturated blue annuli. In contrast, responses from V1 were nearly identical when the annuli were matched for brightness, despite quite different luminance values. This is independent of any individual variations in the luminosity function, which, had this been a complicating factor, would have been as likely to increase the cortical response as to decrease it. Thus, responses at the earliest cortical stages of processing in humans are more consistent with the brightness of a spectral stimulus than its luminance. This suggests that human V1 encodes the underlying similarity in the reflectance values of stimulus sources, rather the luminance values in the corresponding retinal images.

Changing the radiance of the surround of an achromatic target changes the brightness of that target. Correlated with this conscious change in brightness are changes in the activation of cells in V1 in retinotopic register with the target [43]; [27]–[28]. Though consistent with the hypothesis that V1 encodes brightness, such a conclusion is tempered by the fact that the test stimuli used also varied in physical contrast. Here, however, the psychophysical and neuroimaging data presented cannot be accounted for by any changes in the *spatial* context of the stimulus. Rather, it is the *spectral* distribution of each stimulus that provided the necessary context for the spectra's luminance. In other words, the perceptual ‘meaning’ of the luminance signal varied according to its constituent wavelengths, suggesting a direct relationship between luminance and chromaticity processing.

Consistent with this view, three recent, meticulous psychophysical studies revealed strong, non-linear interactions between the human luminance channel and chromatic opponent channels [44]–[46], thus providing the necessary pre-cortical substrate for the correspondence between colour brightness and V1 activity reported here. While arguing for a potential retinal locus for these interactions, these authors also highlighted previous studies showing colour-luminance interactions at the level of single V1 cells [47]–[49]. Together, these and other related studies in other visual qualia [30], [50] suggest that early, non-linear interaction between opponent channels evolved not to encode the physical attributes of stimuli *per se*, but to encode the statistical relationship between stimuli and their past empirical sources, manifesting itself in this case as colour brightness.

### Conclusion

The current study demonstrates that contextual effects on brightness are due to the visual system's attempts to identify the most probable source of ambiguous image data. To do this, it uses the statistical relationship between images (the activation of photoreceptors) and scenes (objects and conditions in the natural world), a relationship encoded in the functional structure of early visual areas.

## Materials and Methods

### Fitting Gaussians to hyperspectral data

For five hyperspectral natural images [51], we randomly sampled 1000 pixels, making a total of 5000 natural spectra, each sampled at 10 nm intervals. For each spectra, we used the Matlab Curve fitting toolbox (v. 1.1) to fit 1, 2 or 3 Gaussian components using a least-squares optimisation. Although there was variation between the scenes, as would be expected, using more components gave a closer fit to the data, with 3 Gaussian components giving a very good fit. Specifically, the models' *r*-squared scores (the coefficient of determination, which measures the proportion of variability in the data accounted for by the model) were typically in the range 0.20–0.56 when using one Gaussian; 0.73–0.91 with two Gaussians; and 0.91–0.96 with three Gaussians. This last class of models produced residual root-mean-squared errors in the range 0.001–0.062, showing that 3 Gaussian components allow the hyperspectral data to be modelled very closely. Note that this is measured independently of any observer.

### Physical correlates of colour properties

**Figure 7:**
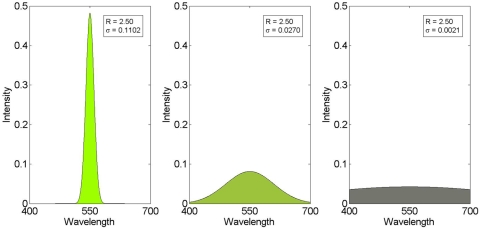
Three example Gaussian spectral power distributions. The standard deviation of the intensity (*y*-axis) of the three curves are the physical correlates of the perceived saturation of the corresponding spectra, with the highest on the left and the lowest on the right. The area under each curve (i.e. the reflectivity) is constant.

The ‘psychophysical’ test set was designed to approximate the kind of stimuli used in typical psychophysical studies, with the requirement that we could sensibly calculate “physical correlates” of perceptual properties of colour, such as hue, saturation and brightness. Figure 7 shows three spectral power distributions (SPD), coloured using an RGB mapping. Each SPD here is defined by a single Gaussian, centred at 550 nm. We used this mean as the physical correlate of hue. All three have the same area under the curve (limited by the range 400–700 nm), as indicated by the constant *R* score. This area is the reflectance, which we used as the physical correlate of brightness, with a constant illuminant. These are equivalent (up to a scaling factor) under a constant illuminant. Finally, the standard deviation of the intensity (on the *y*-axis) of the three curves is shown as *σ*, ranging from 0.11 on the left to 0.0021 on the right. Larger values correspond to a more sharply peaked Gaussian and smaller values to a flatter Gaussian. We therefore used this as a physical correlate of saturation.

### Retinal responses

For each spectral power distribution (SPD) we calculate the human cone responses using known spectral sensitivities [25]. If L(λ) is the sensitivity of the long-wavelength sensitive cone to light with a wavelength of λ, and G(λ) is the amplitude of the SPD at wavelength λ, then the total response of the L-cone is 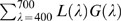, and similarly for the medium- (M) and short- (S) wavelength sensitive cones.

Based on these values, we also calculated the corresponding values of the luminous efficiency function (or luminosity), for use when calculating the constant-luminosity response curves in several figures. Equation 5 in reference [2] gives the energy-based function, equivalent to *V*(*λ*) = 0.64315 *L*(*λ*)+0.39595 *M*(*λ*).

In all cases, we used the energy unit form of the 2° cone fundamentals, using data available from the Colour & Vision database http://cvrl.ucl.ac.uk.

#### Bayesian ideal observer data

All data were generated and analysed using Matlab (v.6.5). To avoid complications with modelling adaptation, and with data on different scales, each variable was independently rescaled to the range zero to one. This range was used for each cone activation function, for the luminosity values and for the reflectance scores. Thus the values used are all relative rather than absolute. For the cone values, this is a form of von Kries adaptation [4].

Each scene is a single uniform surface: any additional surface in the input adds another dimension to the joint probability distribution, which then requires the use of exponentially larger data sets to estimate. For example, if we use a scene of 10×10 = 100 surfaces, each characterised by three cone response values and a reflectance value, the resulting joint distribution has 400 variables. This requires a sample of data in 400 dimensions, and to be representative such as sample would be impracticably large.

#### fMRI subjects and stimuli

Three healthy volunteers (aged 28–35 years) gave written informed consent to participate in the experiments, which were approved by the local ethics committee. Subjects 1 and 2 participated in experiment 1, and subjects 2 and 3 participated in experiment 2. All were neurologically normal and had normal or corrected-to-normal vision. In the first experiment stimuli comprised of three different patterns were presented within a smoothed annular window centred on fixation that subtended from 3° to 7°. The CIE coordinates of the blue standard were Y = 2.6; x = 0.154; y = 0.107); of the equiluminant yellow stimulus were Y = 2.6; x = 0.357; y = 0.438; and of the equally-bright yellow annulus were Y = 3.6; x = 0.362; y = 0.470. In the second experiment, the CIE coordinates of the blue standard were as above; the less-saturated, equiluminant blue were Y = 2.6; x = 0.178; y = 0.150; and the equally-bright blue annulus co-ordinates were Y = 4.1; x = 0.178; y = 0.150.

The pattern stimuli were presented repeatedly in blocks of 20.8 seconds with a presentation time of 500 ms and an inter-stimulus-interval of 540 ms. Each stimulus block was presented twice with randomized sequence and a 20.8 second fixation-only baseline between blocks. To monitor attentiveness, participants were required to maintain gaze on a central fixation spot and perform a simple task that involved detecting a small brightness change at the fixation spot. Stimuli were presented using an LCD projector (NEC LT158) with a frame rate of 60 Hz that projected onto a screen at the head-end of the scanner. This image was viewed through a front-coated mirror with near-flat spectral reflectance profile. The projector was controlled by a graphics card (NVIDIA Quadro4 900 XGL) using Cogent software (http://www.vislab.ucl.ac.uk/Cogent). The spectral properties of the stimuli were individually measured in situ from the screen using a photo-spectrometer (PR-650 SpectraScan). These data were used to provide look-up table for individual coordinates in colour space.

#### *f*MRI acquisition

A Siemens Allegra 3T scanner with standard head coil was used to acquire Blood Oxygenation Level Dependent (BOLD) contrast functional MRI EPI volumes with 32 axial slices at an isotropic resolution of 3×3×3 mm (TR = 2080 ms; TE = 30 ms; flip angle 90°). In experiment 1 ten runs each comprising 126 volumes were acquired per subject. In the second experiment we acquired five runs for subject 2 and four runs for subject 3. For each subject a T1-weighted structural image and standard retinotopic mapping BOLD contrast volumes using meridian stimulation were also acquired.

#### fMRI data analysis

Data were analysed using SPM2 (http://www.fil.ion.ucl.ac.uk/spm) and MrGray [52]. For the retinotopic mapping, to identify V1 we created a mask volume defining this area. This was obtained using the meridian localisers from the retinotopic mapping sessions following standard definitions of V1, together with segmentation and cortical flattening in MrGray. For the main experiment, after discarding the first six images of each scanning run to allow for magnetic saturation effects, the remaining images were realigned, and then co-registered with the structural scans of the individual participants. Data for each of the three stimulation conditions were modelled voxel-wise using a general linear model [53]. This model consisted of box-car regressors for each stimulus type convolved by a canonical hemodynamic response function in SPM2. Regression parameters resulting from analysis of the imaging time series for the main experiment were then extracted for all voxels activated by the annulus in V1, and then divided by the mean signal per run and voxel for conversion to percent signal change

## References

[1] Fairchild MD (1998). Color Appearance Models. 1st ed.

[2] Sharpe LT, Stockman A, Jagla W, Jägle H (2005). A luminous efficiency function, V*(lambda), for daylight adaptation.. J Vis.

[3] Lennie P, Pokorny J, Smith VC (1993). Luminance.. J Opt Soc Am A.

[4] Osorio D, Vorobyev M (2005). Photoreceptor spectral sensitivities in terrestrial animals: adaptations for luminance and colour vision.. Philos Trans R Soc Lond B Biol Sci.

[5] Pridmore RW (2007). Effects of luminance, wavelength and purity on the color attributes: Brief review with new data and perspectives.. Color Research & Application.

[6] von Helmholtz HLF (1867/1924). Treatise on Physiological Optics (1924 Southall translation).

[7] Wyszecki G (1967). Correlate for lightness in terms of CIE chromaticity coordinates and luminous reflectance.. J Opt Soc Am.

[8] Nayatani Y (1998). A colorimetric explanation of the Helmholtz-Kohlrausch effect.. Color Res Appl.

[9] Ayama M, Ikeda M (1998). Brightness-to-luminance ratio of colored light in the entire chromaticity diagram.. Color Res Appl.

[10] Knill DC, Richards W (1996). Perception as Bayesian Inference.

[11] Purves D, Lotto RB (2003). Why we see what we do.

[12] Long F, Purves D (2003). Natural scene statistics as the universal basis of color context effects.. Proc Natl Acad Sci USA.

[13] Howe CQ, Purves D (2005). Natural-scene geometry predicts the perception of angles and line orientation.. Proc Natl Acad Sci USA.

[14] Gurney K (2007). Neural networks for perceptual processing: from simulation tools to theories.. Philos Trans R Soc Lond B Biol Sci.

[15] Corney DPA, Lotto RB (2007). What Are Lightness Illusions and Why Do We See Them?. PLoS Comput Biol.

[16] Geisler WS, Chalupa L, Werner J (2003). Ideal Observer analysis.. The Visual Neurosciences..

[17] Boots B, Nundy S, Purves D (2007). Evolution of visually guided behavior in artificial agents.. Network: Comput Neural Syst.

[18] Regan BC, Julliot C, Simmen B, Viénot F, Charles-Dominique P (2001). Fruits, foliage and the evolution of primate colour vision.. Philosophical Transactions of the Royal Society of London Series B.

[19] Thomson EE, Kristan WB (2005). Quantifying Stimulus Discriminability: A Comparison of Information Theory and Ideal Observer Analysis.. Neural Comp.

[20] Brainard DH, Williams DR, Hofer H (2008). Trichromatic reconstruction from the interleaved cone mosaic: Bayesian model and the color appearance of small spots.. Journal of Vision.

[21] Maloney LT (1986). Evaluation of linear models of surface spectral reflectance with small numbers of parameters.. J Opt Soc Am A Opt Image Sci Vis.

[22] Wachtler T, Lee T, Sejnowski TJ (2001). Chromatic structure of natural scenes.. J Opt Soc Am A Opt Image Sci Vis.

[23] Oxtoby EK, Foster DH (2005). Perceptual limits on low-dimensional models of Munsell reflectance spectra.. Perception.

[24] Foster DH, Amano K, Nascimento SMC, Foster MJ (2006). Frequency of metamerism in natural scenes.. J Opt Soc Am A Opt Image Sci Vis.

[25] Stockman A, Sharpe LT (2000). The spectral sensitivities of the middle- and long-wavelength-sensitive cones derived from measurements in observers of known genotype.. Vision Res.

[26] Bowmaker JK, Heath LA, Wilkie SE, Hunt DM (1997). Visual pigments and oil droplets from six classes of photoreceptor in the retinas of birds.. Vision Research.

[27] MacEvoy SP, Paradiso MA (2001). Lightness constancy in primary visual cortex.. Proc Natl Acad Sci.

[28] Haynes JD, Lotto RB, Rees G (2004). Responses of human visual cortex to uniform surfaces.. Proc Natl Acad Sci USA.

[29] Cornelissen FW, Wade AR, Vladusich T, Dougherty RF, Wandell BA (2006). No Functional Magnetic Resonance Imaging Evidence for Brightness and Color Filling-In In Early Human Visual Cortex.. J Neurosci.

[30] Roe AW, Lu HD, Hung CP (2005). Cortical processing of a brightness illusion.. Proc Natl Acad Sci U S A.

[31] Boyaci H, Fang F, Murray SO, Kersten D (2007). Responses to lightness variations in early human visual cortex.. Curr Biol.

[32] Mullen KT, Dumoulin SO, McMahon KL, Zubicaray GID, Hess RF (2007). Selectivity of human retinotopic visual cortex to S-cone-opponent, L/M-cone-opponent and achromatic stimulation.. European Journal of Neuroscience.

[33] Andrews TJ, Lotto RB (2004). Fusion and Rivalry Are Dependent on the Perceptual Meaning of Visual Stimuli.. Curr Biol.

[34] Lotto RB, Chittka L (2005). Seeing the light: Illumination as a contextual cue to color choice behavior in bumblebees.. Proc Natl Acad Sci USA.

[35] Lotto RB, Wicklein M (2005). Bees encode behaviorally significant spectral relationships in complex scenes to resolve stimulus ambiguity.. Proc Natl Acad Sci USA.

[36] Schlessinger E, Bentley PJ, Lotto RB (2005). Evolving Visually Guided Agents in an Ambiguous Virtual World..

[37] Kaiser PK, Boynton RM (1996). Human color vision.

[38] Lewis A, Zhaoping L (2006). Are cone sensitivities determined by natural color statistics?. J Vision.

[39] Webster MA, Mollon JD (1997). Adaptation and the color statistics of natural images.. Vision Res.

[40] von der Twer T, Macleod DIA (2001). Optimal nonlinear codes for the perception of natural colours.. Network: Comput Neural Syst.

[41] Long F, Yang Z, Purves D (2006). Spectral statistics in natural scenes predict hue, saturation, and brightness.. Proc Natl Acad Sci USA.

[42] Brainard DH, Freeman WT (1997). Bayesian color constancy.. J Opt Soc Am A Opt Image Sci Vis.

[43] Hung CP, Ramsden BM, Chen LM, Roe AW (2001). Building surfaces from borders in Areas 17 and 18 of the cat.. Vision Res.

[44] Stockman A, Plummer DJ (2005). Long-wavelength adaptation reveals slow, spectrally opponent inputs to the human luminance pathway.. J Vis.

[45] Stockman A, Plummer DJ (2005). Spectrally opponent inputs to the human luminance pathway: slow+ L and-M cone inputs revealed by low to moderate long-wavelength adaptation.. J Physiol.

[46] Stockman A, Plummer DJ, Montag ED (2005). Spectrally opponent inputs to the human luminance pathway: slow+ M and-L cone inputs revealed by intense long-wavelength adaptation.. J Physiol.

[47] Lennie P, Krauskopf J, Sclar G (1990). Chromatic mechanisms in striate cortex of macaque.. J Neurosci.

[48] Conway BR (2001). Spatial structure of cone inputs to color cells in alert macaque primary visual cortex (V-1).. J Neurosci.

[49] Johnson EN, Hawken MJ, Shapley R (2004). Cone Inputs in Macaque Primary Visual Cortex.. J Neurophysiol.

[50] Murray SO, Boyaci H, Kersten D (2006). The representation of perceived angular size in human primary visual cortex.. Nat Neurosci.

[51] Foster DH, Nascimento SMC, Amano K (2004). Information limits on neural identification of colored surfaces in natural scenes.. Visual neuroscience.

[52] Wandell BA, Chial S, Backus BT (2000). Visualization and measurement of the cortical surface.. J Cog Neurosci.

[53] Friston KJ, Holmes AP, Worsley KJ, Poline JP, Frith CD (1995). Statistical Parametric Maps in Functional Imaging: A General Linear Approach.. Hum Brain Mapp.

